# Investigating the treatment of vascular risk with simvastatin in secondary progressive multiple sclerosis: analysis from the MS-STAT2 randomized controlled trial

**DOI:** 10.1093/braincomms/fcag240

**Published:** 2026-06-24

**Authors:** Thomas Williams, Nicholas Magill, Nevin A John, Alessia Bianchi, James Blackstone, Marie Braisher, Floriana De Angelis, Alberto Calvi, Sean Apap Mangion, Charles Wade, Marios C Yiannakas, Jonathan Stutters, Antonio Ricciardi, Ferran Prados Carrasco, David MacManus, Claudia A M Gandini Wheeler-Kingshott, Frederik Barkhof, Olga Ciccarelli, Jeremy Chataway, the the MS-STAT2 investigators, Jeremy Chataway, Thomas Williams, Nevin John, Floriana De Angelis, Alberto Calvi, Alessia Bianchi, Sarah Wright, Madiha Shatila, Anisha Doshi, Sean Apap Mangion, Charles Wade, Wallace Brownlee, Claudia A M Gandini Wheeler-Kingshott, Frederik Barkhof, Olga Ciccarelli, Jonathan Stutters, Ferran Prados Carrasco, Antonio Ricciardi, Marios Yiannakas, David MacManus, Mariana Agiu, Vanessa Bassan, Tiggy Beyene, Staci Conway, Batoul Fneich, Ana Herrera Jimenez, Sarah Pullinger, Eirini Samdanidou, Megan Wynne, Marie Braisher, James Blackstone, Rachel Merry, Gil Barton, Leanne Crisp, Josephine Parker, Jennifer Flight, Liz Deane, Chris Frost, Jennifer Nicholas, Stuart Nixon, Judy Beveridge, Sue Pavitt, Siddharthan Chandran, Don Mahad, Peter Connick, Dawn Lyle, Ian Galea, Elisabeth Jarman, Carmen Jacob, Stefania Kaninia, Basil Sharrack, David Paling, Helen Ford, Linford Fernandes, Maruthi Vinjam, Owen Pearson, Gillian Ingram, Christopher Rickards, Marguerite Hill, Nikos Evangelou, Christopher Allen, Abdullah Shehu, Tarunya Arun, Mohamed Belhag, Gavin McDonnell, Fiona Magill, Stephen Ramsay, Ruth Geraldes, Matthew Craner, Daniel Cullen, Helen Birmingham, Ana Cavey, Charles Hillier, Judith Dube, Michelle Davis, Jeban Ganesalingam, Leonora Fisniku, Julia Aram, Julie Newman, Jeremy Hobart, Omar Almasri, Daniel Lashley, Cord Spilker, Outi Quinn, Ruth Bellfield, Seema Kalra, Simon Ellis, Neil Robertson, Emma Tallantyre, Cynthia Butcher, Sreedharan Harikrishnan, Nicola Guckm, Stefano Pluchino, Luca Peruzzotti-Jametti, Miriam Mattoscio, Abhijit Chaudhuri, Elisa Visentin, Grace Fawehinmi, Laura Azzopardi, Timothy Harrower, Clare O’Reilly, Sally Acres, Sarah Statton, Carolyn Young, Roger Mills, Heike Arndt, Martin Lee, Suresh Kumar Chhetri, Mark Maskery, Fayyaz Ahmed, Modar Khalil, David Rog, Victoria Parker, Nimisha Vinod, Eli Silber, George Dervanoulis, Neisha Rhule, Paul Gallagher, Martin Duddy, Lisa Robson, Agne Straukiene, Catherine Marshall, Kathryn Bamforth, Amal Kalil, Richard Nicholas, Leilani Cabreros, Claire M Rice, Thomas Minton

**Affiliations:** Queen Square Multiple Sclerosis Centre, Department of Neuroinflammation, UCL Queen Square Institute of Neurology, Faculty of Brain Sciences, University College London, London WC1N 3BG, UK; Department of Medical Statistics, London School of Hygiene & Tropical Medicine, London WC1E 7HT, UK; Queen Square Multiple Sclerosis Centre, Department of Neuroinflammation, UCL Queen Square Institute of Neurology, Faculty of Brain Sciences, University College London, London WC1N 3BG, UK; Department of Medicine, School of Clinical Sciences, Monash University, Melbourne 3800, Australia; Queen Square Multiple Sclerosis Centre, Department of Neuroinflammation, UCL Queen Square Institute of Neurology, Faculty of Brain Sciences, University College London, London WC1N 3BG, UK; Comprehensive Clinical Trials Unit, Institute of Clinical Trials and Methodology, University College London, London WC1V 6LJ, UK; Queen Square Multiple Sclerosis Centre, Department of Neuroinflammation, UCL Queen Square Institute of Neurology, Faculty of Brain Sciences, University College London, London WC1N 3BG, UK; Queen Square Multiple Sclerosis Centre, Department of Neuroinflammation, UCL Queen Square Institute of Neurology, Faculty of Brain Sciences, University College London, London WC1N 3BG, UK; National Institute for Health and Care Research, University College London Hospitals Biomedical Research Centre, London WC1B 5EH, UK; Queen Square Multiple Sclerosis Centre, Department of Neuroinflammation, UCL Queen Square Institute of Neurology, Faculty of Brain Sciences, University College London, London WC1N 3BG, UK; Queen Square Multiple Sclerosis Centre, Department of Neuroinflammation, UCL Queen Square Institute of Neurology, Faculty of Brain Sciences, University College London, London WC1N 3BG, UK; Queen Square Multiple Sclerosis Centre, Department of Neuroinflammation, UCL Queen Square Institute of Neurology, Faculty of Brain Sciences, University College London, London WC1N 3BG, UK; Queen Square Multiple Sclerosis Centre, Department of Neuroinflammation, UCL Queen Square Institute of Neurology, Faculty of Brain Sciences, University College London, London WC1N 3BG, UK; Queen Square Multiple Sclerosis Centre, Department of Neuroinflammation, UCL Queen Square Institute of Neurology, Faculty of Brain Sciences, University College London, London WC1N 3BG, UK; Queen Square Multiple Sclerosis Centre, Department of Neuroinflammation, UCL Queen Square Institute of Neurology, Faculty of Brain Sciences, University College London, London WC1N 3BG, UK; Queen Square Multiple Sclerosis Centre, Department of Neuroinflammation, UCL Queen Square Institute of Neurology, Faculty of Brain Sciences, University College London, London WC1N 3BG, UK; National Institute for Health and Care Research, University College London Hospitals Biomedical Research Centre, London WC1B 5EH, UK; eHealth Center, Universitat Oberta de Catalunya, Barcelona 08018, Spain; UCL Hawkes Institute, Department of Medical Physics and Biomedical Engineering, University College London, London W1W 7TY, UK; Queen Square Multiple Sclerosis Centre, Department of Neuroinflammation, UCL Queen Square Institute of Neurology, Faculty of Brain Sciences, University College London, London WC1N 3BG, UK; Queen Square Multiple Sclerosis Centre, Department of Neuroinflammation, UCL Queen Square Institute of Neurology, Faculty of Brain Sciences, University College London, London WC1N 3BG, UK; Department of Brain and Behavioural Sciences, University of Pavia, 27100 Pavia, Italy; Queen Square Multiple Sclerosis Centre, Department of Neuroinflammation, UCL Queen Square Institute of Neurology, Faculty of Brain Sciences, University College London, London WC1N 3BG, UK; National Institute for Health and Care Research, University College London Hospitals Biomedical Research Centre, London WC1B 5EH, UK; UCL Hawkes Institute, Department of Medical Physics and Biomedical Engineering, University College London, London W1W 7TY, UK; Department of Radiology & Nuclear Medicine, VU University Medical Centre, Vrije Universiteit Amsterdam, P.O. Box 226600, Amsterdam 1100 DD, The Netherlands; Queen Square Multiple Sclerosis Centre, Department of Neuroinflammation, UCL Queen Square Institute of Neurology, Faculty of Brain Sciences, University College London, London WC1N 3BG, UK; National Institute for Health and Care Research, University College London Hospitals Biomedical Research Centre, London WC1B 5EH, UK; Queen Square Multiple Sclerosis Centre, Department of Neuroinflammation, UCL Queen Square Institute of Neurology, Faculty of Brain Sciences, University College London, London WC1N 3BG, UK; National Institute for Health and Care Research, University College London Hospitals Biomedical Research Centre, London WC1B 5EH, UK

**Keywords:** multiple sclerosis, secondary progressive multiple sclerosis, vascular risk, cholesterol, statins

## Abstract

Vascular comorbidity is associated with more severe disability in multiple sclerosis. However, it is unknown whether treating vascular risk will lead to a disease modifying effect. Given the established role of simvastatin as a modifier of vascular risk, we aimed to investigate whether randomization to simvastatin could mitigate the relationships between serum cholesterol profiles and disease worsening, compared with placebo, in the MS-STAT2 trial. MS-STAT2 (NCT03387670) recruited 964 patients with secondary progressive multiple sclerosis, who were randomized to simvastatin (80 mg) or placebo for 3 years. 246 participants additionally underwent yearly magnetic resonance imaging (MRI). Vascular risks were systematically assessed at trial baseline. In this exploratory analysis, the relationships between cholesterol ratio (total cholesterol/high-density lipoprotein) and longitudinal clinical and MRI outcomes were assessed, comparing simvastatin to placebo groups. At baseline, median (IQR) age was 55 (50–60) years and median cholesterol ratio 3.4 (2.8 to 4.3). 73% were female, and 72% required a walking aid. Higher cholesterol ratio was associated with more severe baseline disability. No longitudinal relationships were observed between cholesterol ratio and clinical or brain atrophy outcomes. However, for each unit increase in cholesterol ratio, T2 lesion volume increased by +2.47% (95% CI: +0.03 to +4.97) from baseline in the placebo group. This relationship was significantly reduced in those randomized to simvastatin (−3.10% [−0.06 to −6.06]). Randomization to simvastatin appeared to mitigate the relationship between higher cholesterol ratios and longitudinal increases in T2 lesion volume in patients with secondary progressive multiple sclerosis. Further multi-modal interventional studies targeting vascular risk in patients with multiple sclerosis are warranted.

## Teaser

Williams *et al.* report that in the MS-STAT2 trial cohort (secondary progressive multiple sclerosis), higher cholesterol was associated with more severe current disability, and longitudinal increases in T2 lesion volume in the placebo group. The relationship between cholesterol and change in T2 lesion volume was significantly reduced in those randomized to simvastatin.

## Graphical Abstract

**Graphical Abstract:**
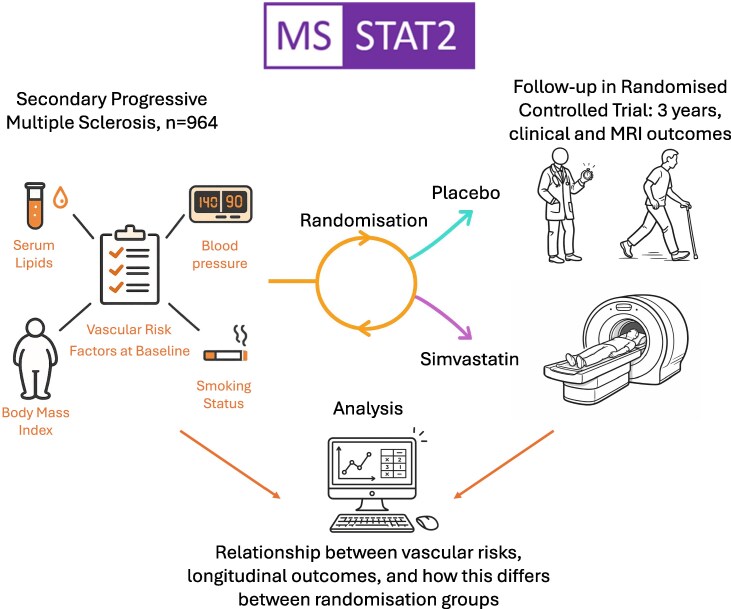

## Introduction

Vascular comorbidities have consistently been associated with more severe disability in patients with multiple sclerosis. This has been demonstrated for smoking, hypercholesterolaemia, raised body mass index (BMI), hypertension, and diabetes.^1-14^ Similar results have been reported for the overall burden of vascular comorbidity, defined using the number of comorbidities or vascular risk scores.^15,16^ Furthermore, people with multiple sclerosis are both at an increased risk of experiencing a vascular event and are more likely to have vascular comorbidities compared with the general population, either at the time of diagnosis or subsequently during the disease course.^17-21^

Given that the various components contributing to an individual’s vascular risk profile are eminently treatable with currently available interventions, it is therefore appealing to consider whether vascular risk modification in itself could be considered as a disease modifying treatment in multiple sclerosis. For example, data from observational studies suggests that those who stop smoking are at lower risk of progression and have reduced brain atrophy compared with those who continue smoking.^22,23^ In the absence of interventional trials targeting vascular risk, however, the extent to which the current observational literature supports a modifiable, causal relationship between vascular risk factors and disability in people with multiple sclerosis is debated.^24^

The MS-STAT2 phase 3 trial (NCT03387670; ISRCTN82598726) randomized almost 1000 patients with secondary progressive multiple sclerosis to high-dose simvastatin (80 mg) or placebo. No therapeutic benefit was seen with simvastatin treatment on any of the primary or secondary clinical outcomes.^25^ Given the well-established role of simvastatin as a modifier of vascular risk, however, the MS-STAT2 trial represents a unique opportunity to investigate whether associations between vascular risk and disease outcomes can be modified with simvastatin treatment. Given that simvastatin principally modifies vascular risk through reductions in cholesterol, we hypothesized that if an adverse cholesterol profile is causally associated with disease worsening, the relationships between participants’ baseline cholesterol levels and subsequent disease worsening will be reduced in those randomized to simvastatin, compared with placebo.^26^ We therefore aimed to assess the relationships between participants’ baseline cholesterol profile, in addition to other vascular risks, and both longitudinal clinical and Magnetic Resonance Imaging (MRI) outcomes, comparing these relationships between simvastatin and placebo groups.

## Materials and methods

### Study design and participants

The MS-STAT2 trial (NCT03387670; ISRCTN82598726) has been described previously.^25^ Briefly, this was a multi-centre, randomized, doubled-blinded phase 3 trial of 80 mg simvastatin once daily, compared with placebo, in patients with secondary progressive multiple sclerosis. Key inclusion criteria were an age of 25 to 65 years, Expanded Disability Status Scale (EDSS) 4.0–6.5 inclusive, and a diagnosis of secondary progressive multiple sclerosis with evidence of recent disability progression. Key exclusion criteria were a diagnosis of primary progressive multiple sclerosis, current use of a statin or Disease Modifying Treatment (DMT) not licenced in the UK for secondary progressive multiple sclerosis, or comorbid type 1 diabetes mellitus. Nested within the main trial, a single-centre MRI sub-study was conducted at the lead University College London Hospital (UCLH) trial site. Eligibility criteria for this sub-study were the same as for the main trial.

The study was conducted in accordance with the Declaration of Helsinki and International Council for Harmonization (ICH) Good Clinical Practice. The National Research Ethics Service Committee (London, Westminster) reviewed the trial protocol and materials to be given to participants (approved 9th October 2017, REC ref 17/LO/1509). All participants provided written informed consent before entering the trial, and separate consent if taking part in the MRI sub-study.

### Vascular risk variables

At screening, all participants underwent a systematic assessment of vascular risks. This included medical history, BMI, smoking status and lipid profiles. Lipid profiles included total cholesterol, low density lipoprotein and high density lipoprotein (HDL). In accordance with current guidelines, lipid profiles were summarized as the cholesterol ratio (total cholesterol divided by HDL).^27^ Patients additionally had their blood pressure (BP) recorded at each trial visit, which was used to determine their mean systolic BP during the trial. Only 5 randomized participants had diabetes mellitus (type 2), hence the modifiable vascular risk factors used in subsequent analyses were cholesterol ratio, BMI, systolic BP and smoking status (never regularly smoked, ex-smoker, current smoker).

### Clinical outcomes

Physical disability was measured with EDSS, Timed 25 Foot Walk (T25FW) and 9-Hole Peg Test (9HPT) at baseline and 6 monthly until 3 years. Information processing speed was assessed with the Symbol Digit Modalities Test (SDMT) at baseline and yearly, and working memory performance was assessed with the Californian Verbal Learning Test-II (CVLT2) and Brief Visuospatial Memory Test-Revised (BVMT-R) at baseline and 3 years.

### MRI analysis

MRI sub-study participants underwent yearly brain MRI on a 3T Philips Ingenia CX MR system from baseline to 3 years. 3D sagittal T1-weighted magnetization ­prepared rapid acquisition gradient echo and 3D sagittal T2-weighted fluid-attenuated inversion recovery images both with 1 mm^3^ isotropic voxels were included. Lesions were automatically segmented via nicMSlesions, quality checked, and then used to derive T2 lesion volumes (T2LV).^28^ These lesion masks were additionally used for lesion filling on the 3DT1 scans.^29^

For cross-sectional analyses, normalized whole brain (WB) and cortical grey matter (cGM) volume was calculated using Structural Image Evaluation using Normalization of Atrophy-X (SIENAX).^30^ Geodesic Information Flows (GIFv2) was also applied to the filled 3DT1 scans to produce brain tissue segmentation and parcellation, which was used to calculate thalamic volumes (adjusted for estimated total intracranial volume).^31^

For longitudinal analyses, the Percentage Brain Volume Change (PBVC) for the whole brain was derived via the Structural Image Evaluation using Normalization of Atrophy (SIENA) method, and for the cGM and thalamus via application of the generalized Boundary Shift Integral (gBSI) method.^30,32^

### Statistical analysis

The presented data are all derived from post-hoc analyses of the MS--STAT2 trial. They were therefore not part of the trial statistical analysis plan, and should be considered exploratory. In accordance with current recommendations, such exploratory analyses will not be adjusted for multiple comparisons, and should be considered hypothesis generating rather than confirmatory.^33,34^

Given the mechanism of action of simvastatin, our analysis focused on baseline cholesterol ratio as the main vascular risk factor, with additional vascular risks (BMI, systolic BP, smoking status) examined in supplementary analyses. In all models, age, sex and (where applicable) trial site were included as covariates.

Ordinal logistic regression models were used to assess the relationship between vascular risk and EDSS at baseline. Longitudinally, Cox proportional hazards models were used with time to EDSS Confirmed Disability Progression (CDP) as the outcome. EDSS CDP was defined as per the trial protocol.^25^ Predictors included the vascular risk factor, randomization group (simvastatin compared with placebo), and the interaction between vascular risk and randomization group.

Linear regression models were used to examine the relationship between vascular risk and T25FW (feet per sec) and 9HPT (sec^−1^ × 1000) at baseline. Longitudinally, logistic regression models were used to assess the odds ratios of CDP (20% worsening) on T25FW or 9HPT by 36 months. Predictors included the vascular risk factor, randomization group, and the interaction between vascular risk and randomization group.

Linear mixed effect models were used to examine the relationships between vascular risk factors and both cognitive and T2 lesion volume outcomes. The dependent variable was the outcome at all available timepoints. Predictors included time, the vascular risk factor, an interaction between time and vascular risk, an interaction between time and randomization group, and a 3-way interaction between time, vascular risk and randomization group. The treatment effect of randomization group was therefore constrained to be zero at baseline, as these assessments were undertaken prior to initiating treatment. This is equivalent to adjusting for baseline scores.^35^ All covariates were included individually and with an interaction with time, and for cognitive outcomes, years in education was also included. A random effect was included at the level of participant, with an unstructured covariance matrix for the residuals to allow for correlation between repeated measures on the same participant. From each model, three results are reported: the relationship between vascular risk factor and outcome at baseline; the relationship between vascular risk factor and change in outcome during follow-up in the placebo group; and the difference in the relationship between vascular risk factor and change in outcome during follow-up in the simvastatin compared with the placebo group. Time was treated categorically, and given existing evidence that simvastatin is unlikely to substantially modify vascular outcomes within a year of initiating treatment, results are presented as the average effect observed from 12 months after randomization (hence the average of the effects at 24 and 36 months for outcomes assessed annually).^36^

The relationships between vascular risk factors and normalized brain volumes (whole brain, cGM and thalami) at baseline were assessed with linear mixed effect models as above. For the longitudinal atrophy analyses, separate linear mixed effects models were used following the methodology outlined by Frost *et al*.^37^ PBVC was first derived for each pair of scans, giving a maximum of 6 PBVC values for participants completing all 4 yearly scans. This data was used as the dependent variable. The exact time interval (in years) between each pair of scans was calculated, and this was included as a predictor, together with the vascular risk factor, randomization group, and the interactions between vascular risk factor and time, treatment group and time, and treatment group, vascular risk factor and time. As previously, covariates included age and sex, and their interaction with time. Each model included a random slope for participant and participant random effects for visit to appropriately allow for the associations between measures that use one of the same MRI scans. As atrophy rates were faster in the first year of follow-up in both groups, different estimates were produced for the first 12 months and the period after this. From these models, the relationship between vascular risk factor and the annualized rate of PBVC for each region of interest in the placebo group are reported, in addition to the difference in this relationship between simvastatin compared with placebo groups. As for cognitive and T2LV outcomes, results are presented as the average effects observed after 12 months.

Data were analysed by intention-to-treat. Given the exploratory nature of the analyses, estimates and 95% confidence intervals (CI) are reported without *P*-values. For all mixed effects models, assumptions and fit were assessed through inspection of the distribution of residuals and residual versus fitted plots. The linearity of the fixed effects were assessed by comparing plots of residuals with predictors. Proportional odds assumptions in ordinal logistic regression were assessed using Wald chi^2^ tests, and partial proportional odds models used where appropriate. In Cox regression models, proportional hazards assumptions were assessed using Grambsch and Therneau tests and log-log plots, resulting in the inclusion of stratification for sex. All analyses were completed in STATA v19.5.

## Results

### Patient characteristics

964 participants with secondary progressive multiple sclerosis were recruited into the MS-STAT2 trial, of whom 246 additionally had data available from the single-centre MRI sub-study. As shown in Table 1, overall trial participants had established secondary progressive multiple sclerosis, with median age 55 years, median multiple sclerosis duration 22 years, and the majority required some form of walking aid at baseline (72% EDSS 6.0 or 6.5).

**Table 1:** Cohort characteristics

		MS-STAT2 cohort	MS-STAT2 MRI sub-study
***n***		964	246
**Age (years)**		55 (50 to 60)	55 (49 to 60)
**Female**		73.0%	75.2%
**Ethnicity**		96.4% white British	93.0% white British
**MS duration (years)**		22.2 (15.9 to 29.5)	23 (16.0 to 30.0)
**SPMS duration (years)**		5.5 (3.5 to 9.3)	6 (4 to 9)
**EDSS:**	4.0 to 5.5:	28.5%	40.2%
	6.0:	36.7%	34.2%
	6.5:	34.8%	25.6%
**Smoking status**	Never smoked:	56.7%	59.8%
	Ex-smokers:	34.0%	25.6%
	Current smoker:	9.2%	14.6%
**Mean systolic blood pressure (mmHg; IQR; range)**		127 (118 to 138; 92 to 171)	127 (119 to 137; 100 to 164)
**Cholesterol ratio (IQR; range)**		3.4 (2.8 to 4.3; 1.5 to 8.1)	3.2 (2.6 to 4.1; 1.5 to 7.4)
**Body Mass Index (kg/m^2^; IQR; range)**		25.3 (22.3 to 29.4; 15.2 to 53.9)	24.2 (21.2 to 27.7; 16.2 to 43.7)

Characteristics of the overall MS-STAT2 cohort, and of the single-centre MRI sub-study. Data are median (interquartile range) unless otherwise stated. MS, multiple sclerosis; SPMS, secondary progressive multiple sclerosis; IQR, interquartile range.

In terms of baseline vascular risk factors, median cholesterol ratio was 3.4, 43% had ever smoked, median systolic BP was 127 mmHg, and median BMI was 25.3 (Table 1).

### Relationships between cholesterol ratio and disease outcomes

Higher baseline cholesterol ratio was associated with more severe baseline disability, with significant relationships observed across lower limb (EDSS and T25FW), upper limb (9HPT) and cognitive (CVLT2 and BVMTR) disability outcomes (Table 2). There was, however, no evidence to suggest that higher baseline cholesterol ratio was associated with future disability worsening in the placebo group, nor evidence to suggest that randomization to simvastatin significantly reduced any relationships between baseline cholesterol ratio and longitudinal clinical outcomes (Table 2).

**Table 2:** Relationship between cholesterol ratio and clinical outcomes

Outcome	Relationship between cholesterol ratio and outcome at baseline		Relationship between cholesterol ratio and longitudinal outcome the in placebo group			Difference in relationship between cholesterol ratio and longitudinal outcome in the simvastatin compared with placebo group		
	OR	Mean difference	OR	HR	Mean difference	Difference in OR	Difference in HR	Difference in mean difference
EDSS (score or CDP)	1.195 [1.071, 1.334]	-	-	0.952 [0.831, 1.091]	-	-	1.109 [0.928, 1.326]	-
9HPT (s^−1^ × 1000 or CDP)	-	−0.601 [−1.176, −0.026]	0.811 [0.562, 1.169]	-	-	1.254 [0.806, 1.950]	-	-
T25FW speed (ft/seconds or CDP)	-	−0.098 [−0.169, −0.027]	0.965 [0.799, 1.166]	-	-	0.937 [0.726, 1.210]	-	-
SDMT (score)	-	−0.602 [−1.357, 0.152]	-	-	0.605 (−0.057, 1.267)	-	-	−0.265 (−1.043, 0.513)
CVLT2 (score)	-	−1.192 [−1.883, −0.502]	-	-	−0.472 [−1.317, 0.372]	-	-	0.035 [−1.141, 1.071]
BVMTR (score)	-	−0.736 [−1.216, −0.256]	-	-	0.475 [−0.159, 1.108]	-	-	−0.410 [−1.233, 0.413]

For EDSS, the relationship between cholesterol ratio and EDSS score at baseline is reported as the OR from an ordinal logistic regression model. Longitudinally, a cox regression model was used to report HR for the relationship between cholesterol ratio and time to EDSS CDP in the placebo group, and separately the difference in this relationship between simvastatin and placebo groups. For 9HPT and T25FW at baseline, the mean difference in test speed (s^−1^ × 1000 for 9HPT, ft/s for 25FW) for a 1 unit increase in cholesterol ratio is derived from a linear regression model. Longitudinal analyses are derived from logistic regression models of 9HPT or T25FW CDP (20% worsening), reporting the OR between cholesterol ratio and CDP in the placebo group, and separately the difference in ORs between simvastatin and placebo groups. For SDMT, CVLT2, and BVMT-R, data are derived from single linear mixed effect models, separately reporting (per 1 unit increase in cholesterol ratio) the mean difference in outcome at baseline, the mean difference in the longitudinal change in outcome from baseline to after 12 months post-randomization in the placebo group, and the difference between simvastatin and placebo groups in the mean difference in the longitudinal change in outcome after 12 months post-randomization. In all models, age, sex, and trial site are included as covariates, with results reported as the estimate [95% confidence interval]. OR, odds ratio; HR, hazard ratio; EDSS, expanded disability status scale; 9HPT, timed 9-hole peg test; CDP, confirmed disability progression; T25FW, timed 25 foot walk; SDMT, symbol digit modality test; CVLT2, Californian verbal learning test-2; BVMTR; brief visuospatial memory test, revised.

**Figure 1:**
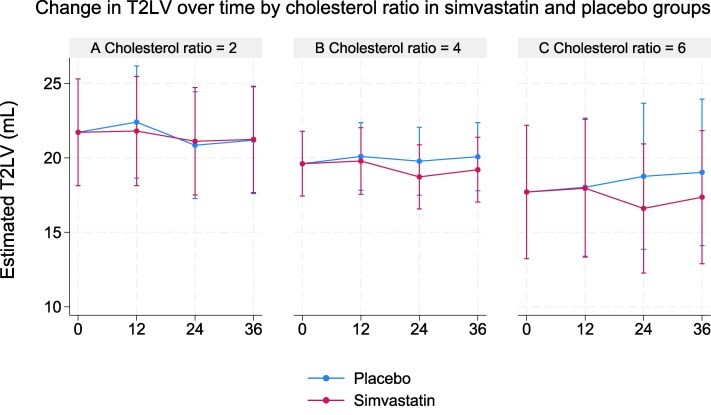
**Change in T2LV over time by cholesterol ratio in simvastatin and placebo groups.** Graphical representation of the data reported in Table 3, modelling the estimated longitudinal changes in T2 lesion volume for the 246 participants of the MS-STAT2 MRI sub-study, comparing the placebo and simvastatin groups, across different baseline cholesterol ratio values. At each timepoint, the point estimate and 95% confidence interval for placebo and simvastatin groups are shown. Linear mixed effect models estimated that in the placebo group, a 1 unit increase in baseline cholesterol ratio was associated with an average 2.47% increase in T2LV from baseline to 24 and 36 months (95% confidence interval: +0.03 to +4.97%). This relationship was significantly reduced after the 12 month timepoint in the simvastatin group, where the increase in T2LV seen with each unit increase in baseline cholesterol ratio was on average −3.10% (95% CI: −0.06 to −6.06%) lower at 24 and 36 months in the simvastatin compared with placebo group. (**A**) baseline cholesterol ratio = 2; (**B**) baseline cholesterol ratio = 4; (**C**) baseline cholesterol ratio = 6. Data are presented after back transformation of log_2_T2 lesion volume values. T2LV, T2 lesion volume.

In the MRI sub-group, baseline cholesterol ratio was not associated with baseline normalized whole brain or regional volumes, nor with subsequent whole or regional brain atrophy. There was, however, a significant relationship between higher baseline cholesterol ratio and a greater increase in T2LV in the placebo group (Table 3). A 1 unit increase in baseline cholesterol ratio was associated with an average 2.47% increase in T2LV from baseline to 24 and 36 months (95% CI: +0.03 to +4.97%) in the placebo group. This relationship was significantly reduced after the 12 month timepoint in the simvastatin group compared with placebo, where the increase in T2LV seen with each unit increase in baseline cholesterol ratio was on average −3.10% (95% CI: −0.06 to −6.06%) lower at 24 and 36 months in the simvastatin compared with placebo group. This difference can be seen in the separation between simvastatin and placebo groups in T2LV after 12 months in those with higher baseline cholesterol ratios (Fig. 1).

**Table 3:** Relationship between cholesterol ratio and MRI outcomes

Outcome	Relationship between cholesterol ratio and outcome at baseline		Relationship between cholesterol ratio and longitudinal change in outcome after 12 months post-randomization		Difference in relationship between cholesterol ratio and longitudinal change in outcome after 12 months post-randomization in simvastatin compared with placebo group	
	Ratios of expected differences	Expected differences	Ratios of expected differences	Expected differences	Ratios of expected differences	Expected differences
T2 lesion volume (% change)	−4.975 [−13.253 to 4.092]	-	2.471 [0.034 to 4.967]	-	−3.104 [−6.055 to −0.061]	-
Whole brain atrophy (mL or PBVC/year)	-	−3.036 [−11.122 to 5.051]	-	0.047 [−0.076, 0.169]	-	−0.012 [−0.183, 0.159]
Cortical grey matter atrophy (mL or PBVC/year)	-	−3.576 [−7.760 to 0.608]	-	−0.019 [−0.117, 0.078]	-	0.060 [−0.079, 0.198]
Thalamic atrophy (mL or PBVC/year)	-	0.083 [−0.058 to 0.223]	-	0.071 [−0.053, 0.194]	-	−0.019 [−0.190, 0.152]

For T2LV, data are reported from a single linear mixed effect model. The relationships between baseline cholesterol ratio and baseline T2LV; between baseline cholesterol ratio and change in T2LV from baseline to after 12 months post-randomization in the placebo group; and the difference in the relationship between baseline cholesterol ratio and change in T2LV after 12 months post-randomization in the simvastatin compared with placebo group are reported. As T2LV was analysed as log_2_(T2LV), data are presented after back-transformation to % changes. For the brain volume models, the relationships between baseline cholesterol ratio and baseline normalized volumes (in mL) are first reported from linear mixed effect models. Separate longitudinal models were then constructed to report the relationship between baseline cholesterol and PBVC after 12 months post-randomization in the placebo group, and the difference in the relationship between baseline cholesterol ratio and PBVC after 12 months post-randomization in simvastatin compared with placebo groups. In all models, age and sex, and their interaction with time, are included as covariates, with results reported as the estimate [95% confidence interval]. PBVC, percentage brain volume change; T2LV, T2 lesion volume.

### Relationships between additional vascular risks and disease outcomes

In supplementary analyses of other vascular risk factors (BMI, mean systolic BP, smoking status; Supplementary Tables 1–6), only smoking was associated with more severe clinical disability at baseline. Current smokers had slower 9HPT speeds, and current or ex-smokers demonstrated poorer cognitive performance (Supplementary Table 5). Higher BMI was associated with smaller normalized whole brain and cGM volumes at baseline, and also with a greater average increase in T2LV from baseline to 24 and 36 months in the placebo group (Supplementary Table 2). In contrast to the result observed for cholesterol ratio, however, the increase in T2LV associated with higher BMI was not significantly different in the simvastatin group compared with placebo (Supplementary Fig. 1).

## Discussion

We have investigated the relationships between baseline vascular risk factors and longitudinal clinical and MRI outcomes over 3 years as an exploratory analysis of a large clinical trial cohort of patients with secondary progressive multiple sclerosis. Moreover, we have utilized the randomization between simvastatin, a known modifier of vascular risk acting primarily via cholesterol reduction, and placebo, to investigate whether the longitudinal relationships between vascular risk factors and outcomes can be modified by simvastatin treatment.

Given the mechanism of action of simvastatin, we particularly focussed on the relationships between baseline cholesterol ratio and disease outcomes. Whilst higher baseline cholesterol was cross-sectionally associated with baseline disease severity on multiple outcome measures, no longitudinal relationships were observed. Higher baseline cholesterol ratio was, however, associated with a greater average increase in T2LV from baseline to 24 and 36 months in the placebo group, a relationship that was significantly mitigated after 12 months post-randomization in patients randomized to simvastatin. In supplementary analyses, a similar relationship was observed for BMI, with higher baseline BMI also being associated with a greater average increase in T2LV from baseline to 24 and 36 months in the placebo group. In contrast to the results with cholesterol ratio, however, randomization to simvastatin did not appear to affect the relationship between BMI and change in T2LV, consistent with the lack of effect simvastatin would be expected to have on BMI.

Our results support the existing literature in demonstrating that adverse vascular risk factors are associated with greater disease severity in people with multiple sclerosis. Adverse lipid profiles have previously been associated with EDSS worsening and smaller brain volumes.^13,14^ The absence of longitudinal relationships between vascular risks and change in clinical disease severity, however, suggests that at this advanced stage of the disease (median multiple sclerosis duration 22 years) and over a relatively short follow-up duration (3 years), such vascular risks did not have a detectable clinically meaningful impact upon disability worsening.

Despite this, however, the observation that baseline cholesterol ratio is associated with subsequent increases in T2LV, a well-validated outcome measure in multiple sclerosis trials, and that this relationship can be mitigated by randomization to simvastatin, does provide support for the hypothesis that hypercholesterolaemia may be a potentially modifiable risk factor for disease worsening.^38^ Interpretation here should, however, be cautious, given the exploratory nature of our analyses. These results should therefore be taken as hypothesis generating rather than confirmatory.^33,34^ Further prospective studies are warranted to investigate the disease modifying potential of vascular risk modification in people with multiple sclerosis, and our analyses have potential implications for the design of such future studies.

Firstly, previous observational studies which have found longitudinal relationships between vascular risks or comorbidity and clinical disability or brain atrophy outcomes have often included participants earlier in their disease course with longer durations of follow-up.^11,12,39,40^ For example, in the pivotal study by Marrie *et al.*,^11^ patients with vascular comorbidities at the time of multiple sclerosis diagnosis (mean age at diagnosis 38 years) required a walking aid after a median of 12.8 years, compared with 18.8 years in those without comorbidity. This could therefore be consistent with the hypothesis that vascular risk factors make a larger contribution to multiple sclerosis pathophysiology earlier in the course of the disease, and that trials aiming to detect clinically meaningful benefits from the treatment of vascular risk may require long durations of follow-up. The mitigation of the relationship between cholesterol ratio and changes in T2LV that we have observed with simvastatin was also only apparent after 12 months of treatment. This is consistent with the results of previous trials investigating prevention of cardiovascular events with simvastatin, where benefits were only observed after 18 months of treatment.^36^ Accommodating the need for prolonged follow-up and such potentially delayed treatment effects may be hard to achieve within typical randomized controlled trial frameworks, but could be considered through registry-based approaches.

Secondly, our intervention (monotherapy with simvastatin) is likely to have had only a moderate impact upon overall vascular risk.^41^ Given simvastatin’s mechanism of action in modifying vascular risk is principally driven by reductions in serum cholesterol, it is unsurprising that the relationship between cholesterol ratio and change in T2LV was modified by randomization to simvastatin, but the relationship between BMI and change in T2LV was not.^26^ If the relationship between BMI and T2LV was separately amenable to treatment (for example, through reductions in BMI with GLP-1 agonists or other interventions), one could hypothesize that complex interventions simultaneously targeting multiple aspects of vascular risk may be more likely to produce clinically meaningful benefits compared with simvastatin alone.^42^

Thirdly, our observation that differences between simvastatin and placebo in the change in T2LV were only observed in those with higher baseline cholesterol ratios suggests that future trials with interventions designed to target vascular risk should particularly recruit participants with adverse vascular risk profiles. This is also consistent with the neutral results of the overall MS-STAT2 trial—demonstrating that simvastatin does not have a clinically meaningful benefit in people with secondary progressive multiple sclerosis that are not selected for higher vascular risk. Indeed, as previously discussed, the overall MS-STAT2 trial cohort may have been at slightly lower vascular risk than the general secondary progressive multiple sclerosis population, due to the exclusion of those already taking a statin.^25^ Patients with multiple sclerosis are known to be at greater risk of vascular events compared with the general population, but the threshold at which interventions should be considered is currently unknown.^17^

The pathophysiological basis for the relationships between cholesterol ratio and BMI with subsequent changes in T2LV in this study is unknown. In older secondary progressive multiple sclerosis cohorts such as MS-STAT2 (median age 55 years), there is likely to be an interaction between multiple sclerosis-related mechanisms and biological ageing.^43^ T2LV is therefore likely to have contributions from both inflammatory multiple sclerosis lesions, and a component of cerebral small vessel disease.^44^ Vascular comorbidities have been associated with increased risk of inflammatory activity in people with multiple sclerosis.^10,15^ Vascular risk factors such as dyslipidaemia and obesity, however, also have established roles in the risk of developing cerebral small vessel disease, and simvastatin is a known modifier of such vascular risks.^45,46^ The apparent mitigation of the relationship between cholesterol ratio and subsequent T2LV changes that we have observed with simvastatin may therefore be due to contributions from both inflammatory and ischaemic pathologies.

The main limitation of this study is its post-hoc design, and hence the outcomes should be considered exploratory. The reliance upon three-way interactions in the statistical modelling also inherently reduces statistical power and limits the precision of our estimates.^47^ It was also deemed unethical to recruit participants into MS-STAT2 if they were at particularly high vascular risk, and patients already taking a statin were excluded. This is likely to have reduced the number of participants with high baseline cholesterol, and contributed to the low prevalence of diabetes in our cohort. Additional potential confounding factors, such as social deprivation and vitamin D levels, were not addressed in this study. Whilst randomization should ensure any such potential confounders do not impact comparisons between simvastatin and placebo groups, we cannot exclude deprivation accounting for some of the relationships seen between vascular risk factors and disease severity at baseline.^48,49^

## Conclusions

In this large RCT cohort of patients with secondary progressive multiple sclerosis, cholesterol ratio was associated with multiple aspects of disease severity at baseline. Despite finding no evidence to support relationships between cholesterol ratio and longitudinal changes in clinical or brain atrophy outcomes at this advanced stage of the disease over 3 years, higher baseline cholesterol ratio was associated with greater increases in T2LV in the placebo group, and randomization to simvastatin significantly reduced this relationship. Further interventional studies targeting multiple aspects of vascular risk as a potential disease modifying approach are warranted in patients with multiple sclerosis.

## Supplementary Material

fcag240_Supplementary_Data

## Data Availability

All data used in this analysis was derived from the MS-STAT2 trial, with examples of the statistical code used provided in Supplementary material. All data requests should therefore be submitted to J.C. for consideration in the first instance. Access to available fully anonymized data might be granted 12 months after publication, after review by J.C. and the sponsor (University College London). Requesters will be asked to complete an application form detailing specific requirements, rationale, and proposed use. A data-sharing agreement will need to be signed. Requested data will be made available, along with supporting documentation (e.g. data dictionary) on a secure server.

## Appendix

List of MS-STAT2 Investigators

Jeremy Chataway, Thomas Williams, Nevin John, Floriana De Angelis, Alberto Calvi, Alessia Bianchi, Sarah Wright, Madiha Shatila, Anisha Doshi, Sean Apap Mangion, Charles Wade; Wallace Brownlee, Claudia A M Gandini Wheeler-Kingshott, Frederik Barkhof, Olga Ciccarelli, Jonathan Stutters, Ferran Prados Carrasco, Antonio Ricciardi, Marios Yiannakas, David MacManus; Mariana Agiu, Vanessa Bassan, Tiggy Beyene, Staci Conway, Batoul Fneich, Ana Herrera Jimenez, Sarah Pullinger, Eirini Samdanidou, Megan Wynne; Marie Braisher, James Blackstone, Rachel Merry, Gil Barton, Leanne Crisp, Josephine Parker, Jennifer Flight, Liz Deane, Chris Frost, Jennifer Nicholas, Stuart Nixon, Judy Beveridge, Sue Pavitt, Siddharthan Chandran, Don Mahad, Peter Connick, Dawn Lyle, Ian Galea, Elisabeth Jarman, Carmen Jacob, Stefania Kaninia, Basil Sharrack, David Paling, Helen Ford, Linford Fernandes, Maruthi Vinjam, Owen Pearson, Gillian Ingram, Christopher Rickards, Marguerite Hill, Nikos Evangelou, Christopher Allen, Abdullah Shehu, Tarunya Arun, Mohamed Belhag, Gavin McDonnell, Fiona Magill, Stephen Ramsay, Ruth Geraldes, Matthew Craner, Daniel Cullen, Helen Birmingham, Ana Cavey, Charles Hillier, Judith Dube, Michelle Davis, Jeban Ganesalingam, Leonora Fisniku, Julia Aram, Julie Newman, Jeremy Hobart, Omar Almasri, Daniel Lashley, Cord Spilker, Outi Quinn, Ruth Bellfield, Seema Kalra, Simon Ellis, Neil Robertson, Emma Tallantyre, Cynthia Butcher, Sreedharan Harikrishnan, Nicola Guck, Stefano Pluchino, Luca Peruzzotti-Jametti, Miriam Mattoscio, Abhijit Chaudhuri, Elisa Visentin, Grace Fawehinmi, Laura Azzopardi, Timothy Harrower, Clare O’Reilly, Sally Acres, Sarah Statton, Carolyn Young, Roger Mills, Heike Arndt, Martin Lee, Suresh Kumar Chhetri, Mark Maskery, Fayyaz Ahmed, Modar Khalil, David Rog, Victoria Parker, Nimisha Vinod, Eli Silber, George Dervanoulis, Neisha Rhule, Paul Gallagher, Martin Duddy, Lisa Robson, Agne Straukiene, Catherine Marshall, Kathryn Bamforth, Amal Kalil, Richard Nicholas, Leilani Cabreros, Claire M Rice and Thomas Minton
