# Supplementary material for: Investigating the treatment of vascular risk with simvastatin in secondary progressive multiple sclerosis: analysis from the MS-STAT2 randomized controlled trial
Source: Brain Commun. 2026 Jun 24;8(4):fcag240. doi: 10.1093/braincomms/fcag240 (PMC13334090; doi:10.1093/braincomms/fcag240)
Supplement: fcag240_Supplementary_Data [file fcag240_supplementary_data.docx]

## Supplementary material:

Supplementary Table 1: Relationship between BMI and clinical outcomes

| Outcome | Relationship between BMI and outcome at baseline | | Relationship between BMI and longitudinal outcome the in placebo group | | | Difference in relationship between BMI and longitudinal outcome in the simvastatin compared to placebo group | | |
| --- | --- | --- | --- | --- | --- | --- | --- | --- |
|  | OR | Mean difference | OR | HR | Mean difference | Difference in OR | Difference in HR | Difference in mean difference |
| EDSS (score or CDP) | 1.010  (0.990 to 1.031) | - | - | 1.001  (0.975 to 1.027) | - | - | 0.992  (0.956 to 1.030) | - |
| 9HPT (s^-1^ x 1000 or CDP) | - | 0.058  (-0.050 to 0.167) | 0.972  (0.909 to 1.040) | - | - | 0.994  (0.910 to 1.086) | - | - |
| T25FW speed (ft/seconds or CDP) | - | -0.009  (-0.022 to 0.004) | 0.973  (0.939 to 1.009) | - | - | 1.002  (0.952 to 1.055) | - | - |
| SDMT (score) | - | 0.093  (-0.049 to 0.235) | - | - | 0.062  (-0.064 to 0.188) | - | - | -0.027  (-0.184 to 0.130) |
| CVLT2 (score) | - | -0.076  (-0.208 to 0.055) | - | - | 0.089  (-0.068 to 0.246) | - | - | -0.141  (-0.360 to 0.079) |
| BVMTR (score) | - | 0.054  (-0.038 to 0.146) | - | - | 0.056  (-0.062 to 0.173) | - | - | -0.063  (-0.224 to 0.098) |

For EDSS, the relationship between BMI and EDSS score at baseline is reported as the OR from an ordinal logistic regression model. Longitudinally, a cox regression model was used to report HR for the relationship between BMI and time to EDSS CDP in the placebo group, and separately the difference in this relationship between simvastatin and placebo groups. For 9HPT and T25FW at baseline, the mean difference in test speed (s^-^1 x 1000 for 9HPT, ft/s for 25FW) for a 1 unit increase in BMI is derived from a linear regression model. Longitudinal analyses are derived from logistic regression models of 9HPT or T25FW CDP (20% worsening), reporting the OR between BMI and CDP in the placebo group, and separately the difference in ORs between simvastatin and placebo groups. For SDMT, CVLT2, and BVMT-R, data are derived from single linear mixed effect models, separately reporting (per 1 unit increase in BMI) the mean difference in outcome at baseline, the mean difference in the longitudinal change in outcome from baseline to after 12 months post-randomisation in the placebo group, and the difference between simvastatin and placebo groups in the mean difference in the longitudinal change in outcome after 12 months post-randomisation. In all models, age, sex, and trial site are included as covariates, with results reported as the estimate [95% confidence interval]. BMI, body mass index; OR, odds ratio; HR, hazard ratio; EDSS, expanded disability status scale; 9HPT, timed 9-hole peg test; CDP, confirmed disability progression; T25FW, timed 25 foot walk; SDMT, symbol digit modality test; CVLT2, Californian verbal learning test-2; BVMTR; brief visuospatial memory test, revised.

Supplementary Table 2: Relationship between BMI and MRI outcomes

| Outcome | Relationship between BMI and outcome at baseline | | Relationship between BMI and longitudinal change in outcome after 12 months post randomisation | | Difference in relationship between BMI and longitudinal change in outcome after 12 months post randomisation in simvastatin compared to placebo group | |
| --- | --- | --- | --- | --- | --- | --- |
|  | Ratios of expected differences | Expected differences | Ratios of expected differences | Expected differences | Ratios of expected differences | Expected differences |
| T2 lesion volume (% change) | -1.796  [-3.771 to 0.219] | - | 0.668  [0.073 to 1.266] | - | -0.020  [-0.744 to 0.709] | - |
| Whole brain atrophy (mL or PBVC / year) | - | -3.587  [-5.305 to -1.869] | - | 0.016  [-0.015 to 0.047] | - | -0.015  [-0.056 to 0.026] |
| Cortical grey matter atrophy (mL or PBVC / year) | - | -2.261  [-3.140 to -1.381] | - | -0.010  [-0.035 to 0.015] | - | 0.013  [-0.020 to 0.046] |
| Thalamic atrophy (mL or PBVC / year) | - | -0.005  [-0.037 to 0.026] | - | 0.007  [-0.025 to 0.038] | - | -0.011  [-0.053 to 0.031] |

For T2LV, data are reported from a single linear mixed effect model. The relationships between baseline BMI and baseline T2LV; between baseline BMI and change in T2LV from baseline to after 12 months post-randomisation in the placebo group; and the difference in the relationship between baseline BMI and change in T2LV after 12 months post-randomisation in the simvastatin compared to placebo group are reported. As T2LV was analysed as log_2_(T2LV), data are presented after back-transformation to % changes. For the brain volume models, the relationships between baseline BMI and baseline normalised volumes (in mL) are first reported from linear mixed effect models. Separate longitudinal models were then constructed to report the relationship between baseline BMI and PBVC after 12 months post-randomisation in the placebo group, and the difference in the relationship between baseline BMI and PBVC after 12 months post-randomisation in simvastatin compared to placebo groups. In all models, age and sex, and their interaction with time, are included as covariates, with results reported as the estimate [95% confidence interval]. BMI, body mass index; T2LV, T2 lesion volume; PBVC, percentage brain volume change.

Supplementary Table 3: Relationship between systolic BP and clinical outcomes

| Outcome | Relationship between systolic BP and outcome at baseline | | Relationship between systolic BP and longitudinal outcome the in placebo group | | | Difference in relationship between systolic BP and longitudinal outcome in the simvastatin compared to placebo group | | |
| --- | --- | --- | --- | --- | --- | --- | --- | --- |
|  | OR | Mean difference | OR | HR | Mean difference | Difference in OR | Difference in HR | Difference in mean difference |
| EDSS (score or CDP) | 0.999  (0.990, 1.008) | - | - | 1.003  (0.993, 1.014) | - | - | 1.011  (0.996, 1.026) | - |
| 9HPT (s^-1^ x 1000 or CDP) | - | 0.037  (-0.008, 0.083) | 0.985  (0.958, 1.012) | - | - | 1.018  (0.983, 1.054) | - | - |
| T25FW speed (ft/seconds or CDP) | - | -0.003  (-0.009, 0.002) | 0.582  (0.234, 1.445) | - | - | 1.007  (0.986, 1.027) | - | - |
| SDMT (score) | - | 0.017  (-0.042, 0.077) | - | - | -0.014  (-0.066, 0.039) | - | - | 0.039  (-0.026, 0.104) |
| CVLT2 (score) | - | 0.012  (-0.043, 0.067) | - | - | -0.044  (-0.110, 0.021) | - | - | 0.077  (-0.014, 0.168) |
| BVMTR (score) | - | 0.002  (-0.036, 0.041) | - | - | 0.017  (-0.033, 0.067) | - | - | -0.015  (-0.083, 0.054) |

For EDSS, the relationship between systolic BP and EDSS score at baseline is reported as the OR from an ordinal logistic regression model. Longitudinally, a cox regression model was used to report HR for the relationship between systolic BP and time to EDSS CDP in the placebo group, and separately the difference in this relationship between simvastatin and placebo groups. For 9HPT and T25FW at baseline, the mean difference in test speed (s^-^1 x 1000 for 9HPT, ft/s for 25FW) for a 1 unit increase in systolic BP is derived from a linear regression model. Longitudinal analyses are derived from logistic regression models of 9HPT or T25FW CDP (20% worsening), reporting the OR between systolic BP and CDP in the placebo group, and separately the difference in ORs between simvastatin and placebo groups. For SDMT, CVLT2, and BVMT-R, data are derived from single linear mixed effect models, separately reporting (per 1 unit increase in systolic BP) the mean difference in outcome at baseline, the mean difference in the longitudinal change in outcome from baseline to after 12 months post-randomisation in the placebo group, and the difference between simvastatin and placebo groups in the mean difference in the longitudinal change in outcome after 12 months post-randomisation. In all models, age, sex, and trial site are included as covariates, with results reported as the estimate [95% confidence interval]. BP, blood pressure; OR, odds ratio; HR, hazard ratio; EDSS, expanded disability status scale; 9HPT, timed 9-hole peg test; CDP, confirmed disability progression; T25FW, timed 25 foot walk; SDMT, symbol digit modality test; CVLT2, Californian verbal learning test-2; BVMTR; brief visuospatial memory test, revised.

Supplementary Table 4: Relationship between systolic BP and MRI outcomes

| Outcome | Relationship between systolic BP and outcome at baseline | | Relationship between systolic BP and longitudinal change in outcome after 12 months post randomisation | | Difference in relationship between systolic BP and longitudinal change in outcome after 12 months post randomisation in simvastatin compared to placebo group | |
| --- | --- | --- | --- | --- | --- | --- |
|  | Ratios of expected differences | Expected differences | Ratios of expected differences | Expected differences | Ratios of expected differences | Expected differences |
| T2 lesion volume (% change) | 0.046  [-0.743 to 0.841] | - | 0.117  [-0.096 to 0.331] | - | 0.094  [-0.183 to 0.373] | - |
| Whole brain atrophy (mL or PBVC / year) | - | -0.270  [-0.989 to 0.449] | - | -0.006  [-0.017 to 0.005] | - | 0.005  [-0.011 to 0.020] |
| Cortical grey matter atrophy (mL or PBVC / year) | - | -0.296  [-0.668 to 0.077] | - | -0.005  [-0.014 to 0.004] | - | 0.003  [-0.010 to 0.016] |
| Thalamic atrophy (mL or PBVC / year) | - | 0.006  [-0.007 to 0.018] | - | -0.007  [-0.019 to 0.004] | - | 0.005  [-0.011 to 0.020] |

For T2LV, data are reported from a single linear mixed effect model. The relationships between baseline systolic BP and baseline T2LV; between baseline systolic BP and change in T2LV from baseline to after 12 months post-randomisation in the placebo group; and the difference in the relationship between baseline systolic BP and change in T2LV after 12 months post-randomisation in the simvastatin compared to placebo group are reported. As T2LV was analysed as log_2_(T2LV), data are presented after back-transformation to % changes. For the brain volume models, the relationships between baseline systolic BP and baseline normalised volumes (in mL) are first reported from linear mixed effect models. Separate longitudinal models were then constructed to report the relationship between baseline systolic BP and PBVC after 12 months post-randomisation in the placebo group, and the difference in the relationship between baseline systolic BP and PBVC after 12 months post-randomisation in simvastatin compared to placebo groups. In all models, age and sex, and their interaction with time, are included as covariates, with results reported as the estimate [95% confidence interval]. Systolic BP, mean systolic blood pressure; T2LV, T2 lesion volume; PBVC, percentage brain volume change.

Supplementary Table 5: Relationship between smoking status and clinical outcomes

| Outcome | Smoking status | Relationship between smoking status and outcome at baseline, compared to non-smokers | | Relationship between smoking status and longitudinal outcome (compared to non-smokers) in the placebo group | | | Difference in relationship between smoking status and longitudinal outcome (compared to non-smokers) in the simvastatin compared to placebo group | | |
| --- | --- | --- | --- | --- | --- | --- | --- | --- | --- |
|  |  | OR | Mean difference | OR | HR | Mean difference | Difference in OR | Difference in HR | Difference in mean difference |
| EDSS (score or CDP) | Ex-smoker | 0.825  (0.641, 1.061) | - | - | 0.992  (0.716, 1.374) | - | - | 0.824  (0.524, 1.296) | - |
|  | Current Smoker | 1.090  (0.730, 1.629) | - | - | 1.044  (0.635, 1.714) | - | - | 0.702  (0.334, 1.475) | - |
| 9HPT (s^-1^ x 1000 or CDP) | Ex-smoker | - | -0.477  (-1.809, 0.854) | 0.256  (0.086, 0.757) | - | - | 2.696  (0.757, 9.595) | - | - |
|  | Current Smoker | - | **-2.445**  **(-4.642, -0.247)** | 1.107  (0.394, 3.111) | - | - | 0.567  (0.113, 2.850) | - | - |
| T25FW speed (ft/seconds or CDP) | Ex-smoker | - | 0.142  (-0.022, 0.306) | 0.841  (0.542, 1.306) | - | - | 1.206  (0.655, 2.220) | - | - |
|  | Current Smoker | - | -0.120  (-0.389, 0.149) | 0.767  (0.390, 1.512) | - | - | 0.795  (0.282, 2.237) | - | - |
| SDMT (score) | Ex-smoker | - | **-1.820**  **(-3.561, -0.080)** | - | - | 0.535  (-1.035, 2.105) | - | - | 0.742  (-1.192, 2.676) |
|  | Current Smoker | - | -2.512  (-5.406, 0.382) | - | - | 0.868  (-1.559, 3.296) | - | - | 0.129  (-2.903, 3.162) |
| CVLT2 (score) | Ex-smoker | - | **-2.126**  **(-3.732, -0.521)** | - | - | -1.005  (-2.968, 0.958) | - | - | 0.582  (-2.112, 3.277) |
|  | Current Smoker | - | **-3.186**  **(-5.835, -0.537)** | - | - | -0.544  (-3.643, 2.554) | - | - | -1.464  (-5.828, 2.899) |
| BVMTR (score) | Ex-smoker | - | -0.202  (-1.330, 0.925) | - | - | -0.742  (-2.235, 0.752) | - | - | 0.781  (-1.229, 2.792) |
|  | Current Smoker | - | -1.849  (-3.726, 0.028) | - | - | -1.089  (-3.378, 1.200) | - | - | 0.603  (-2.585, 3.790) |

For all models, never smokers are taken as the reference to which ex-smokers and current smokers are compared. For EDSS, the relationship between smoking status and EDSS score at baseline is reported as the OR from an ordinal logistic regression model. Longitudinally, a cox regression model was used to report HR for the relationship between smoking status and time to EDSS CDP in the placebo group, and separately the ratio of these HRs between simvastatin and placebo groups. For 9HPT and T25FW at baseline, the mean difference in test speed (s^-^1 x 1000 for 9HPT, ft/s for 25FW) by smoking status is derived from a linear regression model. Longitudinal analyses are derived from logistic regression models of 9HPT or T25FW CDP (20% worsening), reporting the OR between smoking status and CDP (compared to non-smokers) in the placebo group, and separately the ratio of these ORs between simvastatin and placebo groups. For SDMT, CVLT2, and BVMT-R, data are derived from single linear mixed effect models, separately reporting (by smoking status) the mean difference in outcome at baseline, the mean difference in the longitudinal change in outcome from baseline to after 12 months post-randomisation in the placebo group, and the difference between simvastatin and placebo groups in the mean difference (compared to non-smokers) in the longitudinal change in outcome after 12 months post-randomisation. In all models, age, sex, and trial site are included as covariates, with results reported as the estimate [95% confidence interval]. OR, odds ratio; HR, hazard ratio; EDSS, expanded disability status scale; 9HPT, timed 9-hole peg test; CDP, confirmed disability progression; T25FW, timed 25 foot walk; SDMT, symbol digit modality test; CVLT2, Californian verbal learning test-2; BVMTR; brief visuospatial memory test, revised.

Supplementary Table 6: Relationship between smoking status and MRI outcomes

| Outcome | Smoking status | Outcome at baseline, compared to non-smokers | | Change in outcome compared to non-smokers in the placebo group | | Difference in the change in outcome, compared to non-smokers, between simvastatin and placebo groups | |
| --- | --- | --- | --- | --- | --- | --- | --- |
|  |  | Ratios of expected differences | Expected differences | Ratios of expected differences | Expected differences | Ratios of expected differences | Expected differences |
| T2 lesion volume (% change) | Ex-smoker | 20.325  [-4.784 to 52.056] | - | -2.212  [-8.263 to 4.239] | - | 4.137  [-4.294 to 13.312] | - |
|  | Current Smoker | -5.998  [-29.880 to 26.019] | - | 5.207  [-2.744 to 13.808] | - | -1.854  [-11.565 to 8.922] | - |
| Whole brain atrophy (mL or PBVC / year) | Ex-smoker | - | -10.383  [-31.288 to 10.522] | - | 0.130  [-0.209 to 0.469] | - | -0.427  [-0.891 to 0.038] |
|  | Current Smoker | - | 5.031  [-20.860 to 30.921] | - | 0.042  [-0.346 to 0.431] | - | 0.092  [-0.467 to 0.652] |
| Cortical grey matter atrophy (mL or PBVC / year) | Ex-smoker | - | -4.717  [-15.592 to 6.158] | - | 0.038  [-0.237 to 0.314] | - | -0.310  [-0.686 to 0.066] |
|  | Current Smoker | - | 4.253  [-9.222 to 17.728] | - | -0.021  [-0.334 to 0.291] | - | 0.065  [-0.385 to 0.515] |
| Thalamic atrophy (mL PBVC / year) | Ex-smoker | - | -0.150  [-0.514 to 0.214] | - | 0.151  [-0.189 to 0.491] | - | -0.541  [-1.007 to -0.076] |
|  | Current Smoker | - | 0.014  [-0.437 to 0.465] | - | -0.150  [-0.541 to 0.240] | - | 0.049  [-0.511 to 0.610] |

For all models, never smokers are taken as the reference to which ex-smokers and current smokers are compared. For T2LV, data are reported from a single linear mixed effect model. The relationships between baseline smoking status (compared to non-smokers) and baseline T2LV; between baseline smoking status (compared to non-smokers) and change in T2LV from baseline to after 12 months post-randomisation in the placebo group; and the difference in the relationship between baseline smoking status (compared to non-smokers) and change in T2LV after 12 months post-randomisation in the simvastatin compared to placebo group, are reported. As T2LV was analysed as log_2_(T2LV), data are presented after back-transformation to % changes. For the brain volume models, the relationships between baseline smoking status (compared to non-smokers) and baseline normalised volumes (in mL) are first reported from linear mixed effect models. Separate longitudinal models were then constructed to report the relationship between baseline smoking status (compared to non-smokers) and PBVC after 12 months post-randomisation in the placebo group, and the difference in the relationship between baseline smoking status (compared to non-smokers) and PBVC after 12 months post-randomisation in simvastatin compared to placebo groups. In all models, age and sex, and their interaction with time, are included as covariates, with results reported as the estimate [95% confidence interval]. T2LV, T2 lesion volume; PBVC, percentage brain volume change.

Supplementary Figure 1: Change in T2LV over time by Body Mass Index in simvastatin and placebo groups

Graphical representation of the data reported in Supplementary Table 2, modelling the estimated longitudinal changes in T2 lesion volume for the 246 participants of the MS-STAT2 MRI sub-study, comparing the placebo and simvastatin groups, across different baseline BMI values. At each timepoint, the point estimate and 95% confidence interval for placebo and simvastatin groups are shown. Linear mixed effect models estimated that in the placebo group, a 1 unit increase in baseline BMI was associated with an average 0.668% increase in T2LV from baseline to 24 and 36 months (95% confidence interval: +0.073 to +1.266%). This relationship did not significantly differ in the simvastatin compared to placebo group (-0.020%, 95% confidence interval: -0.744 to +0.709). A: baseline BMI = 20; B; baseline BMI = 30; C: baseline BMI = 40. Data are presented after back transformation of log_2_T2 lesion volume values. T2LV, T2 lesion volume; BMI, body mass index.

## Example of statistical coding used to model outcomes:

An example of the statistical code used in the modelling, estimating the relationship between the baseline vascular risk factor, longitudinal outcome measures, and the difference in this relationship between simvastatin and placebo groups, is included below. Modelling was performed in Stata v19.5:

mixed [repeated measures OUTCOME] ///

0.timepoint ///

12.timepoint ///

24.timepoint ///

36.timepoint ///

[baseline vascular risk PREDICTOR] ///

12.timepoint#[baseline vascular risk PREDICTOR] ///

24.timepoint#[baseline vascular risk PREDICTOR] ///

36.timepoint#[baseline vascular risk PREDICTOR] ///

12.timepoint#[RANDOMISATION GROUP] ///

24.timepoint##[RANDOMISATION GROUP] ///

36.timepoint##[RANDOMISATION GROUP] ///

12.timepoint#[baseline vascular risk PREDICTOR] ##[RANDOMISATION GROUP] ///

24.timepoint#[baseline vascular risk PREDICTOR] ##[RANDOMISATION GROUP] ///

36.timepoint#[baseline vascular risk PREDICTOR] ##[RANDOMISATION GROUP] ///

age gender ///

timepoint#age timepoint#gender ///

|| pat_idd: , nocons res(unstructured, t(timepoint)) reml
